# The Implications of Aging Couples’ Linked Lives: Dyadic Associations in Health and Well-Being

**DOI:** 10.1093/geroni/igab046.1147

**Published:** 2021-12-17

**Authors:** Stephanie Wilson, Christina Marini, Amy Rauer

## Abstract

Older adults age in the context of their intimate partnerships. Partners’ lives—their emotions, behaviors, and health—are intricately linked as they navigate the challenges associated with aging. This symposium presents research that illuminates ways partners influence one another later in life. The talks are diverse with regard to their timescale (e.g., years vs. hours) and context (e.g., dementia vs. pain). Dr. Martire will examine associations between declines in one spouse’s physical health over 5 years and the other’s mental health. This talk will further consider whether discussing health concerns exclusively with one’s spouse intensifies such associations. Ms. Nah will show how the pain of both partners (care providers and recipients) contributes to escalating marital conflict over 2 years. Dr. Wilson will demonstrate that emotional reactivity to spousal distress in the lab is associated with increased proinflammatory gene expression up to 80 minutes later, a risky pattern for health if repeated over time. Dr. Monin will examine actor and partner associations of affect and depressive symptoms among people with early-stage dementia and their spouses; the absence of partner associations suggests that emotional spillover may operate differently in early-stage dementia dyads. Dr. Novak will identify correlates of four latent profiles derived from couples’ physical, psychological, and relationship well-being: happy, healthy couples; unhappy, unhealthy couples; and two groups with blissful marriages despite individual problems. Dr. Amy Rauer, an internationally recognized scholar of relationships and health, will discuss ways in which this research advances our understanding of couples’ linked lives.

